# A potential application for life-related organics detection on Mars by diffuse reflectance infrared spectroscopy

**DOI:** 10.1016/j.heliyon.2023.e13560

**Published:** 2023-02-09

**Authors:** Wang Liu, Zhongchen Wu, Wenxi Chen, Guobin Jin, Wei Zhang, Xinfang Lv, Pei Yu, Hong Zhao

**Affiliations:** a School of Space Science and Physics, Institute of Space Sciences, Shandong University, Weihai, Shandong, 264209, China; b Marine College, Shandong University, Weihai, Shandong, 264209, China; c SDU-ANU Joint Science College, Shandong University, Weihai, Shandong, 264209, China; d Research Center for Biological Adaptability in Space Environment, Institute of Space Sciences, Shandong University, Weihai, Shandong, 264209, China

**Keywords:** Life exploration, Mars, Organics, Infrared spectroscopy, Electrostatic discharge

## Abstract

Life information searching is a hot point for Mars exploration. Ancient Mars was very likely to reach a habitable environment, and there was a real possibility of arising life on Mars. However, the current Mars has a harsh environment. Under such conditions, life materials on Mars are supposed to have taken the form of relatively primitive microbial or organic residues, which might be preserved in some mineral matrices. Detection of these remnants is of great significance for understanding the origin and evolution of life on Mars. The best detection method is in-situ detection or sample return. Herein, diffuse reflectance infrared spectroscopy (DRIFTS) was used to detect characteristic spectra and the limit of detection (LOD) of potential representative organic compounds with associated minerals. In view of high oxidation due to the electrostatic discharge (ESD) during dust actives on Martian surface. The degradation of organic matter by ESD process was studied under simulated Mars conditions. Our results show that the spectral characteristics of organic matter are significantly different from that of associated minerals. The different organic samples have different mass loss and color change after ESD reaction. And the signal intensity of infrared diffuse reflection spectrum can also reflect the changes of organic molecules after ESD reaction. Our results indicated that the degradation products of organics rather than organic itself are most likely to be founded on current Martian surface.

## Highlights

• The spectral characteristics of potential Martian organics and simulated Martian minerals were recorded and analyzed.
• LOD of some organic compounds by IR was determined as a reference for life detection on Mars.
• The degradation of organic matter by ESD process was studied to understand the effect of dust storm discharge on the preservation of organic matter on Mars.

## 1 Introduction

Searching for extraterrestrial life is the original driving force of deep space exploration which has been continuously concerned but still has no answer. Currently, the interplanetary exploration indicates that only a few Earth-like bodies are habitable for life in our solar system, such as Mars, comets, and some well-known moons of Jupiter and Saturn [1]. Mars is an Earth-like planet with numerous similarities and characteristics such as geological features and planetary structure [2]. But what is vitally important is that Mars most likely had a habitable environment for life long before Earth [3]. Mars explorations indicate that Mars has all the elements necessary for life, such as: carbon, hydrogen, oxygen, nitrogen, phosphorus [4], life-related catalysts and conditions for life reactions [5].

Mars has the potential to generate life in principle. Mars exploration is divided into three categories: orbital exploration, roving exploration, and *in-situ* exploration [6]. The most precise approach for identification of the mineral/rock composition and element species is *in-situ* exploration. The 1976 Viking expedition conducted four tests on Mars and discovered the presence of benzene, chlorobenzene, chloromethane, and dichloromethane on the Martian surface which were finally unfortunately classified as Earth contaminants [7]. Phoenix discovered highly oxidizing perchlorates in Martian soil in 2008 [8]. The chloromethane and dichloromethane detected by the Viking lander were thought to be possible products of perchlorates reacting with organic molecules in Martian soil. In 2013, Curiosity found dichloralkanes with two to four carbon atoms and even chlorobenzene with six carbon atoms in mudrocks at Columbia Crater [9]. In 2014, Curiosity also found trace amounts of methane near Gale crater providing a new way to look for life on Mars [10]. In 2018, Curiosity used the Sample Analysis at Mars (SAM), a miniaturized analytical chemistry laboratory based on pyrolysis, to characterize the volatile organic chemical composition of near-surface (∼6 cm) sand and rock samples scooped and drilled by Curiosity. SAM has found thiophene, aromatic, and aliphatic compounds in pyrolytic gas from samples collected in 3.5 billion year old sediments in Gale Crater [11]. Currently, Perseverance is also searching for indications of Martian life in Jezero Crater [12]. Water, basic essentials for life, has also been found on Mars, primarily in the form of water vapor in the atmosphere, polar ice deposits, and subterranean water ice, according to the Shallow Radar (SHARAD) on the Mars Reconnaissance Orbiter (MRO) [13]. The ultra-thermal neutron measurements of Mars Odyssey orbiter also revealed substantial volumes of water ice beneath the current Mars [14]. Early histories of Mars and Earth are very similar. Organic matter might be brought to Mars by meteorites or comets since the planet's creation, and reated surface abiogenic or biogenic processes [6]. As a result, it's reasonable to believe that biological materials could exist on Mars.

Given the difficulty of finding Martian microbes, the fact that life forms are scarce and most likely exist in the form of microorganisms or even biological metabolites, and may be enriched in some rocks [15]. For *in-situ* micro-area detection methods like Raman, IR, Laser-induced breakdown spectroscopy (LIBS), and Laser induced fluorescence (LIF), the properties of high concentration and concentrated distribution of organic matter are very suitable for high sensitivity spectral detection [16]. Another benefit of spectral detection is of its quick spectral capture, easy data interpretation, and strong spectral features [17]. Therefore, combining *in-situ* spectroscopic detection of potential microorganisms or metabolites/residual organic chemicals in extraterrestrial bodies is of enormous scientific value.

The species of potential organic matter and their associated mineral are also in questions [18]. According to the current Mars survey, there are many Fe/Mg rich clay minerals on Mars [19,20], such as montmorillonite, which could provide a safe sanctuary for biological materials [21]. Calcium sulfate dihydrate was found as a prevalent mineral in Gale Creater [22,23]. Carbonate has been discovered in the plains of Bellis in the polar region of Mars and Jezero Crater [24] which was also regarded as potential carrier to capture and preserve the organic materials [25].

Cellulose is a glucose-based macromolecule; Chitin is a polysaccharide found in the shells of crustaceans such as low plant fungus, shrimp, crabs, insects, and fungi cell walls; Chitosan is the only natural alkaline polysaccharide known so far, as it is the result of deacetylation of chitin, which has a chemical structure comparable to chitin and cellulose [26]. Amino acids, due to their abiogenic and biological origins, are a valuable target within the family of organics of astrobiological interest [27]. Therefore, the above organic materials were selected as the example to demonstrate the spectral detection ability for exploring living substance.

Because different minerals and organic functional groups produce various vibrational modes, infrared diffuse reflectance spectroscopy (DRIFTS) is selected to characterize the samples in this study which have a wider application in mineralogical and biological studies [28]. Current research has shown that infrared band can accurately and effectively identify the characteristic spectrum of organic molecules [29]. At present, one near-infrared spectrometer (VISIR) and short wave infrared (SWIR) spectrometer were carried by Perseverance rover and Zhurong rover, respectively, to analyze and identify minerals/rocks on the Martian surface of Mars [30,31].

Because the Martian surface environment is very harsh, the external environment factors such as cosmic radiation, high UV radiation and drastic temperature variation would most likely have a serious impact on the storage of microbial metabolites [32]. The presence of large amounts of perchlorates and iron oxide on the Martian surface, hydrogen peroxide in the Martian atmosphere, indicates that the Martian surface environment is very oxidizing [33]. Many studies have been done on the effects of UV radiation on organic degradation. However, dust storms on Mars could make those conditions even worse which might induce the atmospheric discharge and the production of large amounts of perchlorate, making the Martian surface environment even oxidizing [34]. Therefore, investigation of the biological degradation/decomposition related to Martian dust actives under simulated Martian condition is necessary and of important scientific significance. Electrostatic discharge (ESD) refers to the potential discharge phenomenon due to quick particulate collision during Martian dust events. Some active substances (i.e. neutral, ionized, metastable or excited states substances) generated in ESD process can interact with Martian surface materials in various ways and induce some chemical reactions. For example, some active oxygen species (AOS) and oxidizing substances [35] generated by ESD are considered to be an important factor for the removal of organics on the surface of Mars [36]. The degradation effect of CO_2_ glow discharge during ESD on organic matter were investigated [37].

In this study, DRIFTS is used to characterize the potential Martian organic molecules (three natural organic polymers and three amino acids) and their mixtures with Martian simulants. Their spectral features, LOD and component degradation/decomposition related to Martian dust actives are systematically investigated.

## 2 Materials and methods

### 2.1 Samples

Mars Global Simulant (MGS-1) Martian soil simulant (Exolith Lab, Orlando, Florida, USA), montmorillonite k-10 (Al_2_O_9_Si_3_, Macklin), calcium sulfate dihydrate (CaSO_4·_2H_2_O, 99%, HUSHI)，and calcium carbonate (CaCO_3_, 99%, HUSHI) were purchased and used as the associated mineral of target organic matter. Chitin ((C_8_H_13_O_5_N)n, BR, Shanghai yuanyeBio), chitosan ((C_6_HNO_4_)n, 95%, Bide Pharmatech), α-cellulose ((C_6_H_10_O_5_)n, Macklin), and glycine (C_2_H_5_NO_2_, 99.5%, HUSHI), d-alanine (C_3_H_7_NO_2_, 99%, Shanghai yuanyeBio), and l-phenylalanine (C_9_H_11_NO_2_, 99%, Macklin) were chosen as target organic substances. Samples were further grind in an agate mortar for mixing before each experiment. The components of MGS-1 are mainly based on data gathered by Curiosity at Gale Crater [38]. For better illustrative purposes, the target organic compounds were further subdivided into two classes: amino acids and natural organic compounds (i.e., Non-amino acids component chitin, chitosan and cellulose).

### 2.2 The diffuse reflectance infrared fourier transform spectroscopy (DRIFTS)

The DRIFTS was performed using a Bruker spectrometer, model Vertex 70, coupled to a diffuse Reflection accessory (EasiDiff, PIKE). The spectral region is from 400 to 4000 cm^−1^, with a resolution of 4 cm^−1^. Samples spectra and background were average value with an accumulation of 256 scans per spectra using pure aluminum mirror coated with SiO_2_ protective film as standard reflecting plate. After the target powder was loaded into the sample cup during every measurement, the sample surface used the spatula to shave, with the aim of measuring accurately. Finally, the spectra were recorded with atmospheric compensation and the baseline correction, using the software ‘Opus 6.0’ (BRUKER, Germany).

### 2.3 The Mars chamber and ESD device

The ESD normal glow discharge experiment was performed in a Mars chamber under simulated Martian conditions, which was reported by Wu et al. (2018) [34]. A vacuum pump was used to extract the air pressure to vacuum (∼10 Pa) before the experiment began for excluding the interference of air, and then desiccated CO_2_ atmosphere (99,9%, Haohui chemical Co., LTD, Yantai, China) was pumped to the chamber to maintain the atmospheric pressure at 3.0 ± 0.1 mbar. The sample was placed between two copper plate electrodes with a diameter of 35 mm and a distance of 6.0 mm in the Martian chamber. The discharge voltage of low temperature plasma excitation power supply (model: CTP-2000K, Corona Lab, Nanjing, China) was kept at 120 V, and the current was 0.22 mA.

## 3 Results and discussion

### 3.1 Spectral characteristics of pure organic matter

In this study, DRIFTS was used to record and analysis the spectral feature of pure organics, representative Martian minerals and their mixtures in order to investigate its identification and detection ability.

**Fig. 1:**
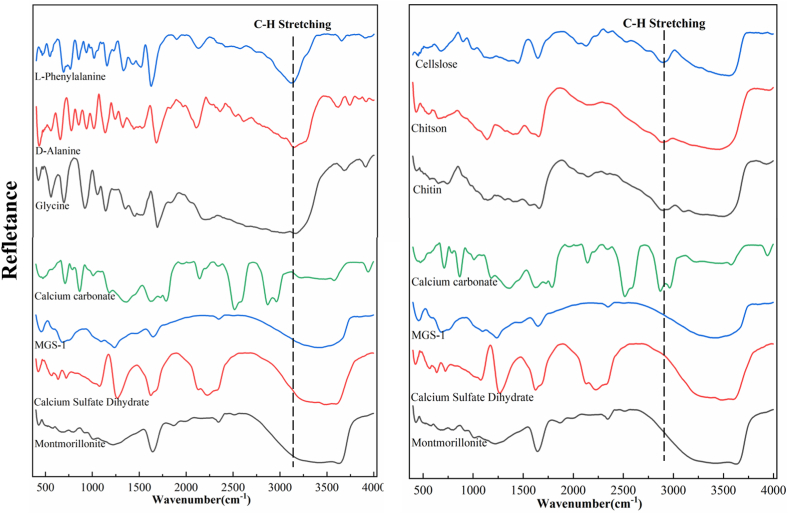
FTIR Diffuse reflection of pure natural organic materials, amino acids and Martian minerals.

The DRIFTS of pure organic compounds and Martian mineral simulants showed rich spectral bands (Fig. 1). All spectra were smoothed using Origin 2021b version (OriginLab, USA), with window points of 50 and polynomial order of 3. As shown, natural organic compounds displayed typical absorption bands at 2800-2900 cm^−1^, and amino acids appeared at 3100-3200 cm^−1^. Both bands can be assigned to C–H stretching vibrations [39], and strictly distinguished from the spectra of Martian mineral simulants. Although pure calcium carbonate exhibits two distinct absorption bands near 2900 cm^−1^ (CO_3_^2−^ vibration bands), it does not prevent it from being distinguished from organic matter [40].

### 3.2 Limit of Detection（LOD）of various organics by DRIFTS

LOD is an important index of detection ability which depends on both analysis method and target mixture. In this study, LOD of mixed sample (listed in Table 1) was determined by DRIFTS.

**Table 1:** LOD of various organics in Martian simulants (wt%). note: “-” means the LOD is more than 40 wt%.

Simulated Martian minerals				
Organic Compounds	MGS-1 (wt%)	Montmorillonite (wt%)	Calcium sulfate dihydrate (wt%)	Calcium carbonate (wt%)
Chitin	2	15.5	2.5	–
Chitosan	0.5	8	1	–
Cellulose	1	–	7	20.5
d-Alanine	11	5.5	–	2
Glycine	–	–	–	–
l-Phenylalanine	10.5	10.5	–	10.5

As observed, in MGS-1 mixture, the LOD of natural organic molecules (0.5 wt %-2.0 wt %) were much lower than of the amino acids (10.5 wt %-11.0 wt %). The latter was hard to be detected in calcium sulfate dihydrate mixture, but much easier to be detected in calcium carbonate mixture. It should be noted that glycine is difficult to be detected in all four mineral matrices. In all, natural organic molecules are easier to detect than amino acids. Therefore, this study shows that DRIFTS is a promising technology for *in-situ* detecting part of organic molecules on Mars. Some organic matter can encounter some limitations by IR detection such as glycine in this study.

### 3.3 Components decomposition by ESD

This experiment mainly studied the color and spectral change of different organic materials along various ESD reaction time. Different pure organic powders were used as precursors which were filled in a fused quartz cell (18 mm in diameter, 0.5 mm in depth) for ESD reaction under simulated Mars conditions (99.9%, CO_2_, 3 mbar).

**Fig. 2:**
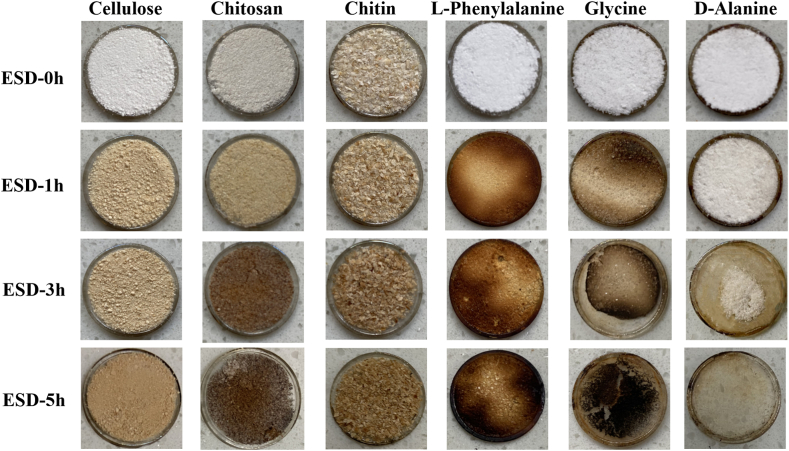
Colors changes or sample reduction of different organic compounds after various ESD reaction. (For interpretation of the references to color in this figure legend, the reader is referred to the Web version of this article.)

ESD reaction strongly impacts on the organic compounds. With the reaction of ESD in Mars chamber, the samples surface changed from original color to brown or yellow, and the color changed more significantly along with the reaction time (Fig. 2). Another interesting phenomenon was that the amount of organic matter lost after ESD reaction. This might be caused by the decomposing of some organic molecules and their products in the form of volatilization gas by CO_2_ glow discharge reactions conditions.

**Fig. 3:**
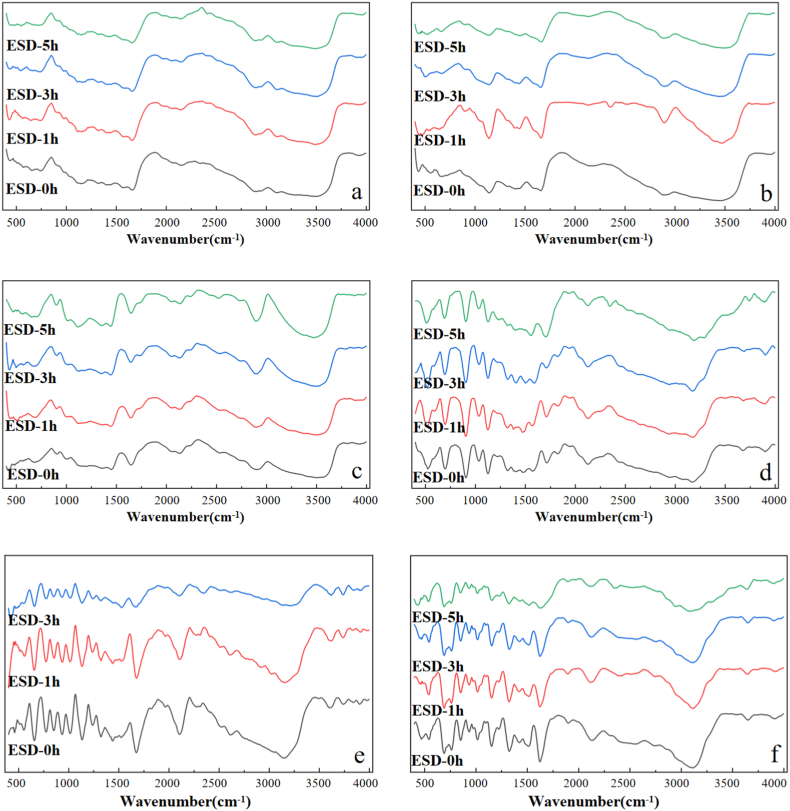
FTIR spectra of pristine pure organic compounds (a. Chitin, b. Chitosan, c. Cellulose, d. Glycine, e. d-Alaine, f. l-Phenylalanine.) and ESD reaction for 1,3,5 h.

Although the obvious color change was observed, the DRIFTS spectra of pure organic compounds after 1,3,5 h ESD plasma reaction under simulated Mars conditions still exhibited all the spectral features (no new absorption peaks) of pristine organic compounds (Fig. 3). Only subtle spectral differences were observed. For instance, in Fig. 3 a-b and e-f, the intensity of all the absorption peaks and the peak profile became more shallower and gradually weaker along with the ESD reaction time which indicated that those peaks should completely disappear after long enough reaction time. This might be caused by the change of color, morphology change of surface, or amorphous caused by sample decomposition under glow condition.

However, cellulose (Fig. 3 c) was an exception whose absorption peak intensity increased along with reaction time which might be due to the other newly generated organic matter during CO_2_ discharge conditions. On the other hand, we also speculated that the change of sample color would hinder the direct irradiation of CO_2_ plasma reaction on amino acid during ESD reaction, and slow down its degradation to a certain extent [41]. If true, the degradation rate is slower than that of amino acids.

In particular, an obvious carbonization was observed on the surface of glycine and l-phenylalanine after short time (1 h) ESD reaction, and the degree of blackening increased with the reaction time. Carbonization might be produced from organic samples instead of from CO_2_ atmosphere, because, as a comparison, no obviously carbonization was observed on the face of d-alanine after 5 h of ESD reaction when it was completely degraded.

Our results show that both the color and spectral feature of organic samples were changed, especially the amino acids, after long-term ESD reaction. In fact, organic compounds on the Martian surface can also be decomposed by the high levels of UV radiation, gamma rays, energetic protons of the sun and galactic cosmic rays (penetrating to several meters of Martian soils) [42]. These effects must be considered in future Martian data interpretation. Moreover, the water, eolian, chemical and other weathering processes together with many ancient unknowns make the life information even difficult to be detected. Based on the above analysis, primitive organic matter on Mars would be hard to be detected under the current Mars surface environment. And the degradation or new reaction products are the key components for Martian life exploration. Ancient lakebed sediments may be the right place to search for life.

## 4 Implications for the detection of life on Mars

*In-situ* analysis is essential to understanding the fate of organic matter on the Martian surface. The current laboratory studies primarily focus on the impact of UV radiation on organic matters. The effect of Martian dust event discharge on organic signals is rarely studied. And previous researches mainly use Raman spectroscopy and infrared absorption spectroscopy to analyze the organics. Due to the advantages of DRIFTS with freedom from interference and no sample preparation, it was selected for the first time to characterize the reaction products of organic targets after ESD reaction.

As reported, the infrared spectra of organic molecules decreases significantly along with reduction of mass and color change by long time of UV radiation, which would ultimately be responsible for the total degradation of the IR signal [[41], [42], [43]]. Those phenomenons are similar and consistent with our results of organic molecules by ESD reaction. UV radiation, galactic cosmic rays and glow conditions were also reported as a catalyst or energy source to form new chemical bonds in organic samples [[37], [38], [39], [40], [41], [42], [43], [44]]. However, no similar phenomenons are observed in our studies. Paul et al. (2010) showed that plasma induced by ESD in dust storm on Mars is likely to generate highly reactive species, and the reaction speed and intensity should be significantly higher than that of UV radiation [37]. Compared to previous studies, our findings show that the ESD reaction has a greater degradation ability of organic materials within less time.

Before getting real Martian samples from the Mars Sample Return (MSR) missions to analysis, *in-suit* detection is still the most effective tool for life-information detection on Mars. The simulation experiment in laboratory is also necessary for building new method or interpreting of the Martian *in-suit* exploration data. In this study, DRIFTS was used to record and analysis characteristic spectra and the LOD of representative organics with associated minerals. Our results demonstrate that DRIFTS is a feasible *in-situ* detection tool for potential Martian organics.

## 5 Conclusions

Searching for life information on Mars surface is critical for progressive discussion on Mars exploration. In this work, the spectral features and LOD of potential representative organic compounds are obtained by DRIFTS. Despite the LOD of various organic compounds is a little higher, the Martian life forms should possibly grow and reproduce in the form of colonies where high amounts of organic matter should be significantly enriched and easily detectable. Therefore, choosing the right detection site is also very crucial. For the first time, we used DRIFTS to experimentally characterize the degradation of organic matter in CO_2_ glow discharge, and found a certain regularity along with the reaction time by DRIFTS. It is valuable for Mars mission to search life information on Mars. Our results also prove that DRIFTS is an effective tool for the identification of potential life markers on Mars.

## Author contribution statement

Wang Liu, Zhongchen Wu: Conceived and designed the experiments; Performed the experiments; Analyzed and interpreted the data; Wrote the paper.

Wenxi Chen, Guobin Jin: Performed the experiments; Analyzed and interpreted the data.

Wei Zhang, Xinfang Lv, Pei Yu, Hong Zhao: Contributed reagents, materials, analysis tools or data, Wrote the paper.

## Funding statement

This work was supported by the National Natural Science Foundation of China (U1931211,42173045), the Preresearch project on Civil Aerospace Technologies No. D020102 funded by China National Space Administration (CNSA) and the Fundamental Research Funds for Shandong University (2019ZRJC007).

## Data availability statement

Data will be made available on request.

## Declaration of interest’s statement

The authors declare no conflict of interest.
